# Screening for effective cell-penetrating peptides with minimal impact on epithelial cells and gut commensals *in vitro*

**DOI:** 10.3389/fphar.2022.1049324

**Published:** 2022-11-02

**Authors:** Hitesh P. Gelli, Ruben Vazquez-Uribe, Morten Otto Alexander Sommer

**Affiliations:** Bacterial Synthetic Biology Group, Novo Nordisk Foundation Center for Biosustainability, Technical University of Denmark, Kongens Lyngby, Denmark

**Keywords:** cell-penetrating peptides, cytotoxicity, permeation enhancer, cell viability, gut microbiota, intestinal absorption, caco-2 cells, *in vitro*

## Abstract

One of the biggest challenges for oral drug absorption is the epithelial barrier of the gastrointestinal tract. The use of cell-penetrating peptides (CPPs) to modulate the epithelial barrier function is known to be an effective strategy to improve drug absorption and bioavailability. In this study we compare side-by-side, 9 most promising CPPs to study their cytotoxicity (Cytotox Red dye staining) and cell viability (AlamarBlue staining) on epithelial cells and their effects on paracellular permeability of the intestinal barrier *in vitro* in a differentiated Caco-2 epithelial monolayer model. The data revealed that 4 out of 9 well-studied CPPs significantly improved Caco-2 paracellular permeability without compromising on cellular health. To assess the impact of CPPs on the human microbiota we studied the antimicrobial effects of the 4 effective CPPs from our permeation studies against 10 representative strains of the gut microbiota *in vitro* using microbroth dilution. Our data revealed that these 4 CPPs affected the growth of almost all tested commensal strains. Interestingly, we found that two synthetic CPPs (Shuffle and Penetramax) outperformed all the other CPPs in their ability to increase intestinal paracellular permeability at 50 µM and had only a small to moderate effect on the tested gut commensal strains. Based on these data Shuffle and Penetramax represent relevant CPPs to be further characterized *in vivo* for safe delivery of poorly absorbed therapeutics while minimizing negative impacts on the gut microbiota.

## Introduction

Over the past decades, several proteins and peptides have been developed as biotherapeutic agents for the treatment of various diseases (Hoofnagle & Di Bisceglie, 1997; Hirsch, 2005; Wang et al., 2022). Even though oral administration is preferred, most of the therapeutic proteins and peptides are administered parenterally (Eek et al., 2016). Oral delivery is challenging for therapeutic proteins and peptides because of low stability caused by the acidic pH, proteolytic enzymes in the gastrointestinal (GI) tract and limited absorption from the GI tract into the systemic circulation due to barriers formed by the mucus and epithelial cell layers (Goldeberg & Gomez-Orellana, 2003; Drucker, 2020). The intestinal epithelial lining is a crucial biological barrier, which is regulated by tight junctions, that are made up of proteins including occludins, claudins and zonula occludens (ZO-1 and ZO-2) (Chelakkot et al., 2018; Ali et al., 2020). Tight junctions are responsible for closing intercellular gaps, thereby determining the paracellular permeability and epithelial barrier integrity (Johnson & Quay 2005; Deli, 2009). To overcome these permeability challenges for improving drug uptake, several strategies have been explored, including the use of medium chain fatty acids (Izgelov et al., 2020), biosurfactants (Perinelli et al., 2017), ingestible capsules (Abramson et al., 2019) and cell-penetrating peptides (CPPs) (Kristensen and Nielsen, 2016a; Rehmani and Dixon, 2018; Diedrichsen et al., 2021).

CPPs are amphipathic and cationic peptides consisting of 5–30 amino acids (Patel et al., 2019). CPPs are often categorized into three types, based on 1) Origin (synthetic, protein-derived, and chimeric CPPs); 2) Conformation (linear and cyclic CPPs); and 3) Physical-chemical properties (cationic, hydrophobic, and amphipathic CPPs) (Xie et al., 2020). CPPs are highly diverse and exhibit different physicochemical and biological properties (Kauffman et al., 2015). CPPs are commonly used to enable cellular intake or translocation of themselves or a CPP-drug/peptide conjugate by promoting permeation across the cellular plasma membrane in the context of drug delivery. However, for the trans-epithelial delivery of polypeptide drugs, paracellular route might be more suitable due to two reasons: 1. Lower proteolytic activity and 2. Paracellular spaces are aqueous filled channels through which these drugs prefer to diffuse (Salamat-Miller and Johnston, 2005; Patel and Misra, 2011). Several studies have reported that some CPPs can also promote permeation of various cellular barriers through paracellular route both *in vitro* and *in vivo* (Ohtake et al., 2002; Bai et al., 2008; Kristensen and Nielsen, 2016a; Kristensen and Nielsen, 2016b; Kristensen et al., 2020; Diedrichsen et al., 2021; Ragupathy et al., 2021; Frøslev et al., 2022). This could be either due to the high local concentration of the CPPs, influencing the dynamics of the tight junction proteins or due to cell-penetrating tendency of CPPs, targeting intracellular proteins which are involved in regulation of opening and closing of tight junctions (Taverner et al., 2015; Kristensen and Nielsen, 2016a). Due to these characteristics, CPPs have been investigated as permeation enhancers in the context of drug delivery coupled to different cargoes such as peptides, proteins, nucleic acids, nanoparticles, and drug molecules (Xie et al., 2020; Kurrikoff et al., 2021).

Transactivating transcriptional activator (Tat) was the first CPP discovered from Human Immunodeficiency Virus 1 (HIV-1). Since then, more than 1,500 CPPs have been identified or synthesized and most of these are defined in the manually curated CPP database (CPPsite 2.0) (Agarwal et al., 2016; Liu et al., 2022). Besides their application as intracellular and paracellular permeation enhancers, CPPs have also been studied for their antimicrobial properties against several pathogenic bacteria and viruses (Pärn et al., 2015; Bocsik et al., 2019; Buccini et al., 2021). In that context, CPPs which seem to exhibit antimicrobial effects on pathogens, may have an impact on commensal gut microbes as well. In recent years, a significant amount of evidence has emerged indicating that the gut microbiome has an important role in human health (Fan and Pedersen, 2021; Vijay and Valdes, 2021), as well as progression of metabolic disorders, cardiovascular diseases, and brain disorders (Turnbaugh et al., 2009; Sampson and Mazmanian, 2015; Bauer et al., 2016; Tang et al., 2017). Therefore, disruption of the gut microbiome may have significant effects on the host’s health. For instance, oral administration antibiotics has been shown to affect the composition of the endogenous microbiota (Li et al., 2019; Ait Chait et al., 2020; Ramirez et al., 2020; Seelbinder et al., 2020; Kang et al., 2021), leading to major complications in health (Kelly & LaMont, 2008; Dethlefsen & Relman, 2011; Neuman et al., 2018). Similarly, CPPs have been studied in the context of antimicrobial peptides and demonstrated to have antimicrobial effects in various studies (Palm et al., 2006; Jung et al., 2008; Bocsik et al., 2019). Co-administration of CPPs *in vivo* is required in large concentrations of a mM range (Kamei et al., 2008). It is thus safe to assume that these CPPs might also have an impact on the gut microbiome. Therefore, it is important to study *in vitro* the impact of CPPs on gut commensals before these peptides are applied in a pre-clinical or clinical setting.

Despite broad applicability of CPPs, there are not many studies comparing the ability of CPPs to enhance paracellular permeability of the intestinal barrier or their potential toxic effects on intestinal cell viability. Furthermore, there are not many reports on the antimicrobial effects of the CPPs against gut commensal microbes. The purpose of this study was to compare 9 of the most well characterized CPPs (Table 1) side-by-side in terms of their effects on the integrity of the epithelial intestinal barrier and toxicity on epithelial cells and further to study the antimicrobial effects of the most effective CPPs based on the permeation studies, against multiple representative species of the gut microbiota*.* We chose 8 out of 9 CPPs based on their successful employment as vectors for *in vitro* transepithelial or *in vivo* delivery of peptide and protein cargoes as summarized by (Kristensen & Nielsen, 2016b). In addition, PN159 was picked due to its ability to permeate Caco-2 monolayers through the paracellular route by effective modulation of tight junctions at low concentrations as reported by Bocsik et al in 2019.

**TABLE 1:** Amino acid sequences of the various cell-penetrating peptides used in this study.

CPP name	Amino acid sequence	Origin	References
Tat	YGRKKRRQRRR	Protein derived	Green and Loewenstein, (1988)
RRL helix	RRLRRLLRRLRRLLRRLR	Synthetic	Kamei et al. (2008)
R9	RRRRRRRRR	Synthetic	Mitchell et al. (2000)
Shuffle	RWFKIQMQIRRWKNKK	Engineered	Kamei et al. (2013)
R8	RRRRRRRR	Synthetic	Mitchell et al. (2000)
pVEC	LLIILRRRIRKQAHAHSK	Protein derived	Elmquist and Langel, (2003)
Penetratin	RQIKIWFQNRRMKWKK	Protein derived	Derossi et al. (1994)
Penetramax	KWFKIQMQIRRWKNKR	Engineered	Kamei et al. (2013)
PN159 (or) KLAL (or) MAP	KLALKLALKALKAALKLA-amide	Synthetic	Oehike et al. (1998)

A, alanine; F, phenylalanine; G, glycine; H, histidine; I, isoleucine; K, lysine; L, leucine; M, methionine; N, asparagine; Q, glutamine; R, arginine; S, serine; W, tryptophan; Y, tyrosine.

## Results

### Effects of CPPs on epithelial cell toxicity and viability

**FIGURE 1:**
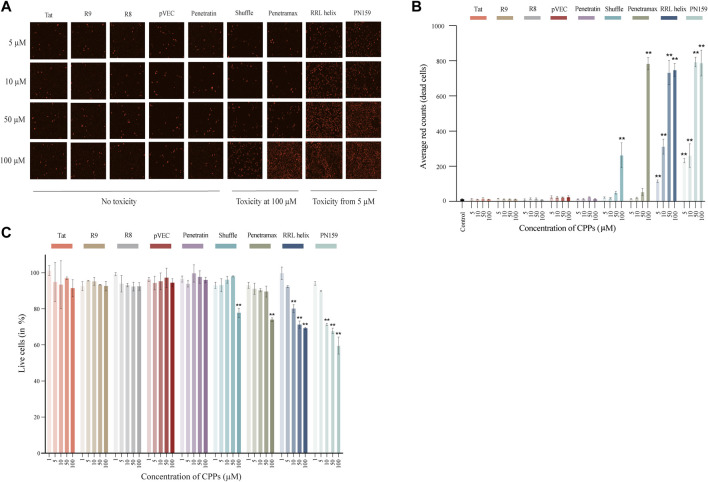
Cell toxicity and viability of Caco-2 cells treated with different CPPs. **(A)** Images of Cytotox Red dye stained Caco-2 cells after 1 h treatment with CPPs. **(B)** Quantification of average red counts (dead cells) of Cytotox Red dye stained Caco-2 cells using Incucyte standard analysis. **(C)** AlamarBlue assay after 1 h treatment with different CPPs. The % live cell values given have been normalized to the control wells (considered as 100% viable). Values are represented as means ± SD, *n* = 2 (cytotoxicity assay) and *n* = 3 (cell viability assay). Statistical analysis: Analysis of variance (ANOVA) followed by Dunnett’s multiple comparisons test, *p* < 0.01 as compared to the control group.

First, we assessed the effects of different concentrations of CPPs on the viability of intestinal epithelial cells to determine a concentration for each of the CPPs that has no impact on cellular health. To determine that, two types of assays were carried out using human epithelial Caco-2 cell lines treated with CPPs: 1) Cytotox Red dye staining was performed to visually differentiate between dead and live cells, 2) AlamarBlue staining was carried out to quantify the percentage of live cells after CPP treatment. Following the treatment of Caco-2 cells with CPPs, we observed three types of cytotoxicity outcomes: no evident toxic effect, highly toxic at 5 µM concentrations or a dose-response effect (Figures 1A,B). The majority of the CPPs (Tat, R8, R9, pVEC, and Penetratin) did not exhibit an evident cytotoxic effect on the Caco-2 cells at any of the tested concentrations. RRL helix and PN159 were toxic at 5 µM and continued to increase in toxicity with higher concentrations in a dose-response manner. Shuffle and Penetramax exhibited significant toxicity only at the highest concentration tested (100 µM). Furthermore, we accurately quantified the percentage of viable cells after CPP exposure (Figure 1C) using the AlamarBlue cell viability assay. Results from the AlamarBlue assay were concordant with the cytotox Red dye staining except that the CPPs RRL helix and PN159 seemed to have no significant toxic effect at 5 µM. From these assays, we determined a concentration for each CPP at which no major cytotoxicity or effects on cell viability were observed.

### Effect of CPPs on epithelial barrier integrity

**FIGURE 2:**
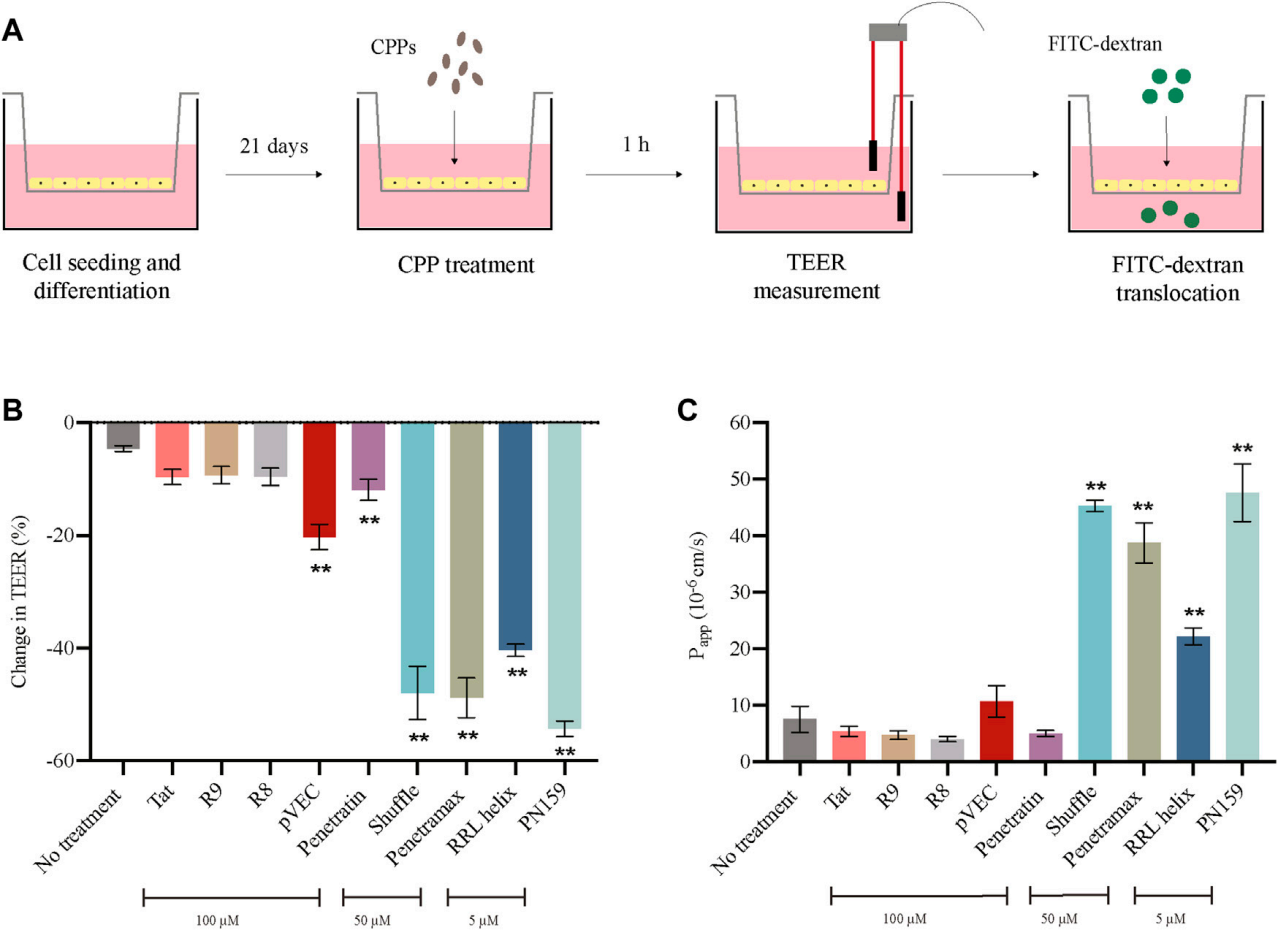
Effect of CPPs on barrier integrity of intestinal epithelial cells. **(A)** Pictorial representation of TEER and FITC-dextran translocation assays. **(B)** Change in TEER values after 1 h treatment with CPPs. **(C)** Permeability of FITC-dextran (Papp A-B 10^-6 cm/s) after 1 h treatment with CPPs. Values are presented as means ± SD, *n* = 3. Statistical analysis: Analysis of variance (ANOVA) followed by Dunnett’s multiple comparisons test, *p* < 0.01 as compared to the control group (no treatment).

After determining a concentration at which no effects were observed on cytotoxicity and cell viability for each of the CPPs, we evaluated the effect of the CPPs on the barrier integrity of the epithelial monolayers. To do this, we measured the 1) transepithelial electrical resistance (TEER), and 2) translocation of Fluorescein Isothiocyanate labelled dextran (FITC-dextran) (Figure 2A). If the CPPs can modulate the tight junctions of the epithelial monolayers, we expect a reduction in TEER as well as an increase in FITC-dextran translocation from apical to basolateral side after treatment with CPPs. Most of the tested CPPs caused reduction in the TEER values of the Caco-2 monolayers after 1 h treatment (Figure 2B). Amongst these, RRL helix, Penetratin, Shuffle, pVEC, Penetramax, and PN159 caused significant reduction in TEER values, indicating high permeability of the epithelial barrier. In agreement with this, the same CPPs except for pVEC and Penetratin showed the highest paracellular permeability (passage from apical to basolateral side) of FITC-dextran (Figure 2C). As RRL helix, Shuffle, Penetramax, and PN159 seem to be most effective in improving intestinal permeability, these CPPs were selected to further study their impact on gut commensal microbes.

### Effect of CPPs on gut microbial strains

**FIGURE 3:**
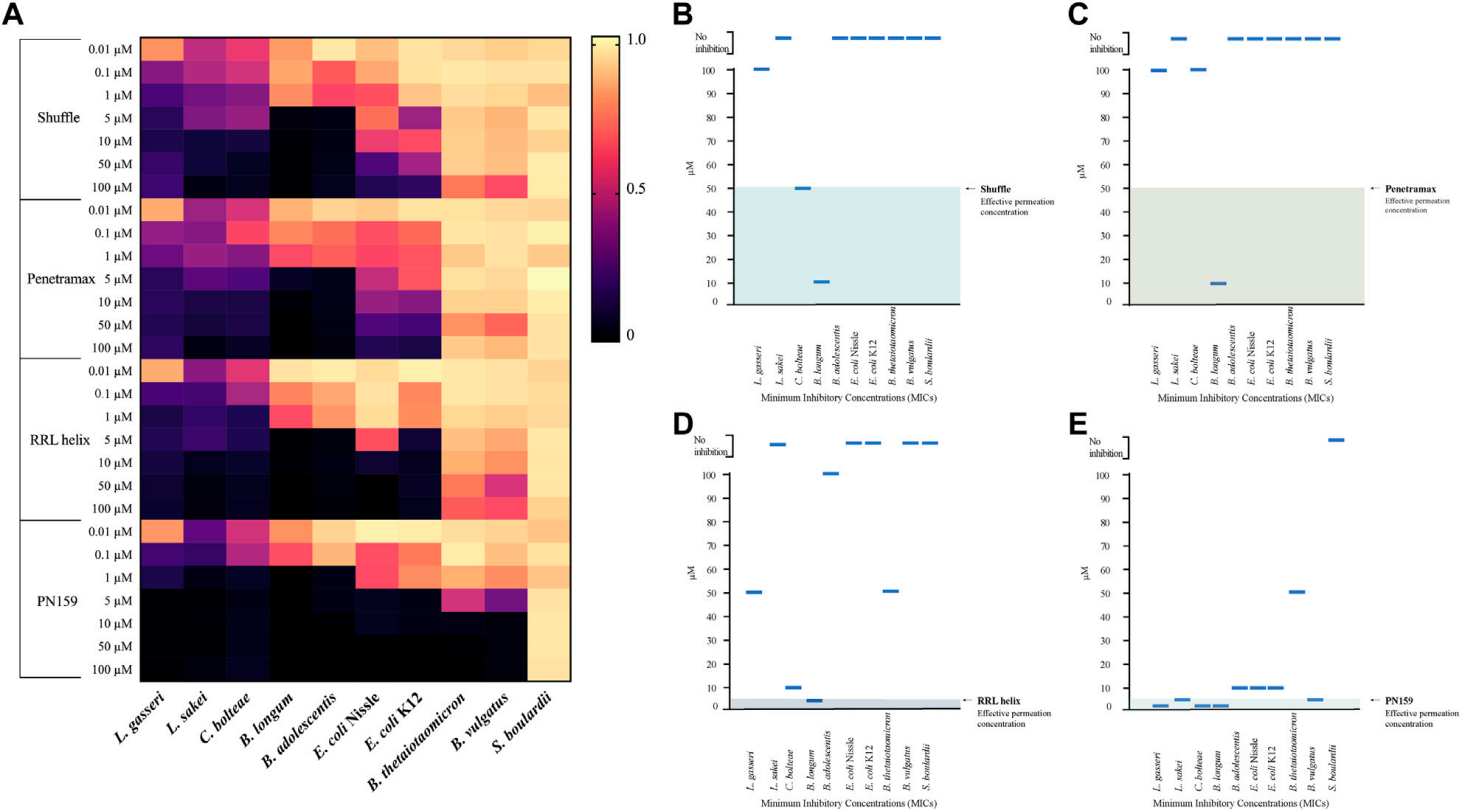
Antimicrobial effects of CPPs on gut commensal microbial strains. **(A)** Heat map showing the antimicrobial effects of the CPPs RRL helix, Shuffle, Penetramax, and PN159 in various concentrations ranging from 0.01 µM to 100 µM against different commensal gut microbial strains. The final OD_600_ values after 24-hour treatment with CPPs were normalized to values between 0 and 1, where 1 being full growth (no CPP treatment) and 0 being no growth (media only). **(B–E)** showing the minimum inhibitory concentrations (MICs) along with their effective permeation concentrations (from permeability study) for the CPPs Shuffle, Penetramax, RRL helix, and PN159 respectively.

The gut microbiome is an important component of the GI tract. However, in previous CPP studies little attention is given to effect of these peptides on commensal microbes. Therefore, we decided to test the anti-microbial activity of the most effective CPPs from barrier integrity assays against 10 commensal gut microbial strains (Table 2). The microbes tested include species from the most abundant phyla in the gut microbiome: Bacteroidetes (*Bacteroides vulgatus* and *Bacteroides thetaiotaomicron*)*,* Firmicutes (*Lactobacillus gasseri, Latilactobacillus sakei, and Clostridium bolteae*)*,* Actinomicetya (*Bifidobacterium longum*, and *Bifidobacterium adolescentis*) and Proteobacteria (*Escherichia coli* Nissle 1917 and *Escherichia coli* K12). In addition, we included a strain of *Saccharomyces boulardii* to evaluate the effect on yeast. Strains were cultured in aerobic or anaerobic conditions in incremental concentrations of the CPPs (0, 0.01, 0.1, 1, 5, 10, 50 and 100 µM). Growth was determined by measuring the optical density of the cultures 24 h post-treatment and MICs were calculated for the CPPs against all the strains (Supplementary Table S1) and are represented in Figures 3B–E. All four tested CPPs displayed a dose-dependent antimicrobial effect (Figure 3A). However, some strains were more sensitive than others, for instance the Firmicutes strains tested were on average more sensitive than the rest of the strains. Concentrations as low as 0.01 µM affected the growth of *L. gasseri, L. sakei* and *C. bolteae* of the CPPs tested. In contrast, the Bacteroidetes species tested were able to tolerate higher concentrations of the CPPs. *B. thetaiotaomicron* and *B. vulgatus* were able to tolerate most CPP concentrations for RRL helix, Shuffle, and Penetramax but were totally inhibited by PN159 (MIC = 10 µM). PN159 showed the most inhibitory effect against almost all the tested strains, where some being inhibited at concentrations as low as 1 µM (Figure 3E). RRL helix exhibited total inhibition (Figure 3D) against half of the tested strains (MIC = 5–100 µM) and a partial inhibitory effect on the remaining strains. Shuffle and Penetramax showed only moderate total inhibitory effects (Figures 3B,C) on most of the tested strains. Interestingly, *S. boulardii* was the most resistant strain against all the four CPPs as its growth was not affected even at 100 µM by any of the CPPs tested.

**TABLE 2:** List of the different gut commensal strains used in this study.

Phylum	Strain name	Gram stain
*Bacillota (Firmicutes)*	*Lactobacillus gasseri* DSM 20077	Positive
	*Latilactobacillus sakei* ATCC 15521	Positive
	*Clostridium bolteae* DSM 29485	Positive
*Actinomicetya*	*Bifidobacterium longum* ATCC 15697	Positive
	*Bifidobacterium adolescentis* DSM 20083	Positive
*Proteobacteria*	*Escherichia coli* Nissle 1917 (Mutaflor)	Negative
	*Escherichia coli* K12 MG 1655	Negative
*Bacteroidetes*	*Bacteroides thetaiotaomicron* DSM 2079	Negative
	*Bacteroides vulgatus* ATCC 8482	Negative
*Ascomycota*	*Saccharomyces boulardii* ATCC MYA-796	Not applicable

## Discussions

Cell penetrating peptides (CPPs) are promising tools to enhance absorption of different drugs, including oral delivery of therapeutic proteins and peptides. However, CPPs have diverse mechanisms of membrane permeation, which are difficult to predict form their physical properties: sequence, molecular weight, and charge (Oliveira et al., 2021). CPPs have been explored for their application as permeation enhancers to improve absorption of drugs through the epithelium of the GI tract, therefore, it is highly possible that these CPPs might interact with the gut microbiota in the GI tract. Some studies have pointed out that some CPPs can exhibit antimicrobial activity against bacteria, including pathogens. However, studies involving CPPs often focus on intracellular uptake and transcellular transport but have not studied their potential enhancement of paracellular transport, which is more suited for transport of oral polypeptide drugs. In regard to toxicity of CPPs, most studies focus on toxicity on host cells but not on their effects on gut microbiota. It is therefore important to experimentally evaluate and compare their paracellular permeability, potential toxicity against host’s cells and gut microbiota to get a better mechanistic understanding, effectiveness, and safety profiles of the CPPs.

In the present work we systematically screened for effective CPPs that promote intestinal permeation through paracellular route and have negligible or no harmful effects against human epithelial cells and gut microbial strains. We observed that CPPs Tat, R8, R9, pVEC, and Penetratin did not have an apparent toxic effect on epithelial cells (Figure 1B), but at the same time, these CPPs did not exhibit paracellular permeability in the concentrations tested as seen in TEER and FITC-dextran assays collectively (Figures 2B,C). Tat and Penetratin are among the most studied CPPs, which have been employed as a vector to improve intracellular delivery of various drug compounds, including oligonucleotides (Brown & Graham, 2010) and proteins (Harada et al., 2002; Liang & Yang, 2005; Kamei et al., 2008; Khafagy et al., 2009). Besides that, both Tat and Penetratin have been shown to promote permeation through tight junction modulation (Bai et al., 2008; Kristensen et al., 2020; Diedrichsen et al., 2021). However, results from our study showed that Tat and Penetratin had no permeabilizing effect in the Caco-2 monolayer setup at 100 µM. More recent findings by **Diedrichsen et al** in 2021 demonstrated that Penetratin, at 60 µM did not improve paracellular permeability of Caco-2 monolayers. Oligoarginine peptides R8 and R9 have been shown to be effective tools at improving intestinal absorption of therapeutic peptides, without damaging the epithelial barrier integrity in rats (Kamei et al., 2008; Morishita et al., 2008; Bocsik et al., 2019). In one study, polyarginine (R5) was shown to improve paracellular permeability of the nasal epithelium (Ohtake et al., 2002). However, we did not observe reduction in TEER or elevated FITC-dextran translocation in the case of R8 and R9 at 100 µM in our experiments, which is in accordance with the results from a previous study (Bocsik et al., 2019), where R8 did not have an influence on the barrier integrity of Caco-2 cells even at 100 µM. This discrepancy in observations could be due to the following reasons: 1) difference in concentrations of CPPs used, 2) differences in experimental setup (*in vitro* vs. *in vivo*), and 3) differences in CPPs administration (CPP-drug conjugate vs. co-administration).

In contrast, RRL helix, Shuffle, Penetramax, and PN159 significantly increased the paracellular permeability of the epithelial barrier when tested in concentrations that did not impact cellular health *in vitro* (Figures 2B,C). The results from the cytotoxicity and cell viability assays were in concordance except for RRL helix and PN159 at 5 µM. This minor difference most likely has to do with the sensitivity of the assay. The reducing capacity of the cells (as measured by AlamarBlue) is not the same as membrane integrity (as measured by Cytotoxic Red). RRL helix significantly improved both the TEER and FITC-dextran translocation, indicating paracellular permeability of the Caco-2 monolayers at 5 µM in this study. This CPP has been previously shown to successfully improve insulin absorption and bioavailability in rats (Kamei et al., 2008). Shuffle is a sequence optimized analogue of Penetratin and has been shown to have an improved drug delivery potential, which could be due to the rearrangement of hydrophobic tryptophan residues as it has been demonstrated that tryptophan residues in an amphipathic CPP sequence positively impact on the internalization into cells (Madani et al., 2011). In a study in 2013, Kamei et al further optimized the sequence of Shuffle to synthesize multiple analogues. Out of these analogues, Penetramax significantly improved the intestinal delivery of insulin compared to Shuffle. Furthermore, **Diedrichsen et al** showed in 2021 that both Shuffle and Penetramax could reduce the TEER of the Caco-2 monolayers and improve paracellular permeability of FITC-dextran, thereby altering the epithelial barrier integrity at 60 μM, like the TEER and FITC-dextran data from our results (Figures 2B,C). Finally, PN159 caused the highest reduction in TEER values and a drastic increase in FITC-dextran translocation, thereby exhibiting the highest paracellular permeability at 5 µM. These results are comparable to the results reported by **Bocsik et al** in 2019 where PN159 was shown to significantly improve paracellular permeability of Caco-2 monolayers *via* tight junction modulation at 3 µM. Based on their potent effect on paracellular permeability and low toxicity, we chose RRL helix, Shuffle, Penetramax, and PN159 to further evaluate their effect on representative microbial species from the gut microbiome.

We tested the antimicrobial effects of the four best CPPs from our permeability studies and found that all the CPPs had an antimicrobial effect against most of the gut microbial strains used in this study. The strains *L. gasseri*, *L. sakei*, and *C. bolteae* seemed to be most affected by the CPPs as their growth was affected either partially by all the CPPs in concentrations as low as 0.01 µM, then followed by *B. longum* and *B. adolescentis* strains, which were mildly affected in the lowest concentrations but were totally inhibited at slightly higher concentrations (MICs = 1 and 10 µM respectively). It is evident from the results that Gram-positive strains are more susceptible to CPPs than Gram-negative strains. This might be because Gram-negative bacteria have an outer cell membrane, which makes them more resistant to antimicrobials, while Gram-positive bacteria lack this layer (Miller, 2016; Exner et al., 2017; Gupta et al., 2019). *S. boulardii* was resistant to all the four CPPs even at the highest concentrations of 100 µM. Similarly, Palm et al in 2006, tested the antimicrobial effects of pVEC, Penetratin, and PN159 against *Saccharomyces cerevisiae* and found no inhibition in concentrations up to 25 µM. These results indicate that the CPPs might be active only against bacteria but less or not active against yeast. However, this can only be concluded after testing these CPPs against multiple yeast strains. PN159, which had a strong permeability effect, exhibited the strongest antimicrobial effect, inhibiting the growth of most strains as shown in Figure 3E (MICs = 1–50 µM). It is the only CPP that significantly inhibited the growth of both *B. vulgatus* (MIC = 10 µM) *and B. thetaiotaomicron* (MIC = 10 µM). Previous studies have shown that PN159 can inhibit pathogens like *Pseudomonas aeruginosa* and *Staphylococcus aureus* at concentrations lower than 50 µM (Bocsik et al., 2019) and E. coli K12 at 25 µM (Palm et al., 2006), while in our study, the growth inhibition was already seen at 5 µM. This variation could be due to the differences in media used, strains tested or the experimental setup.

PN159 significantly improved paracellular permeability of Caco-2 cells and showed no significant effect on cell viability at 5 µM. However, due to its strong antimicrobial effects at its effective permeation concentration (Figure 3E), it might not be ideal for use as a permeation enhancer. RRL helix showed total inhibition only against 1 strain in its effective permeation concentration (Figure 3D). At the same time, the CPP did not cause high permeability of FITC-dextran as compared to Shuffle, Penetramax, or PN159. Possibly, a high dose of this peptide is required to improve paracellular permeability, but that might be toxic to the epithelial cells and cause more damage to the microbiome. Finally, Shuffle and Penetramax showed significant improvement in paracellular permeability in the TEER and FITC-Dextran assays without having significant effects on cell toxicity and viability at 50 µM. Looking at both partial (Figure 3A) and total (MICs) inhibitory effects (Figures 3B,C) of these CPPs, they have a relatively smaller antimicrobial effect in comparison to PN159. Therefore, from this study, considering toxicity to host cells, permeation enhancement effects, and antimicrobial activity of tested CPPs, we suggest that CPPs Shuffle and Penetramax might be suitable to use as permeation enhancers for oral drug delivery through the paracellular route.

Of note, the results in this study are from *in vitro* experiments, therefore it is important to evaluate these CPPs in pre-clinical or clinical studies for a better understanding of their effects on intestinal permeability and safety profiles. The *in vitro* trans-well membrane setup contains only a layer of differentiated epithelial cells and lacks essential factors or barriers of the intestine like the mucus layers or the gut microbiota. These components are essential for maintenance of the epithelial barrier integrity and to carryout absorption by the intestine (Jalili-Firoozinezhad et al., 2019; Gieryńska et al., 2022). Furthermore, the anti-microbial activity was tested against only 10 representative gut commensal strains. In contrast, animals and humans have a much more complex GI tract, which is under constant exposure to a multitude of stimuli and the epithelial surface is in close contact with the mucus layer and is inhabited by billions of microorganisms (Gieryńska et al., 2022). Further research should focus on evaluating these CPPs in more complex in vitro models like gut-on-a-chip (Jalili-Firoozinezhad et al., 2019), where it would be possible to co-culture stable microbial communities with epithelial cells in anaerobic conditions.

In this study, we have highlighted the importance of evaluating the effects of CPPs on both target human cells and commensal gut microbes. These *in vitro* data provide a basis for selection of CPPs for further characterization *in vivo* with a goal to identify CPPs that safely and effectively enhance absorption of oral therapeutics specifically *via* the paracellular route in addition to the already known transcellular route, with minimal impact on the gut microbiome.

## Materials and methods

### Peptides

All peptides (Table 1) were synthesized by Genscript. Stock peptide solutions were prepared to have a final concentration of 1 mM and were stored at -20°C in aliquots. Working concentrations of the peptides were prepared in appropriate mammalian cell culture or bacterial growth media for each experiment.

### Working peptide concentrations

Stock solutions of all the CPPs were prepared in sterile distilled water at a concentration of 1 mM. Treatment solutions were made up of Dulbecco’s Modified Eagle Medium (DMEM) without phenol red (Sigma-Aldrich). Final working concentrations of the peptides in the treatment solutions were as follows: 1, 5, 10, 50, and 100 µM in cell cytotoxicity and barrier integrity assays. In addition to these concentrations, 0.1 and 0.01 µM concentrations were included for antimicrobial assays.

### Cell culture and maintenance

Human intestinal epithelial Caco-2 cell line was purchased from ATCC (catalog number: HTB-37). Caco-2 cells were grown in DMEM cell culture medium supplemented with 10% fetal bovine serum (FBS) (Gibco; Thermo Fisher Scientific, Inc.), 1% MEM Non-Essential Amino Acids (NEAA) (Gibco; Thermo Fisher Scientific, Inc.), and 1% penicillin-streptomycin (Thermo Fisher Scientific) in an incubator at 37 °C with 5% CO_2._

### Caco-2 cell viability and cytotoxicity assays

Caco-2 cells were seeded (6 × 10^3^ cells/well) in Corning™ Costar™ 96-Well, Cell Culture-Treated, Flat-Bottom Microplate and grown at 37°C with 5% CO_2_ until reaching confluence. For alamarBlue cell viability staining, cells were exposed to treatments (DMEM + AlamarBlue (10 µL) + CPPs) at different concentrations and incubated for 2 h at 37°C with 5% CO_2._ Fluorescence was measured with microplate reader Synergy™ H1 BioTek; (excitation wavelength: 560 nm, emission wavelength: 590 nm) and a curve of Relative Fluorescence Units (RFU) against different concentrations of CPPs was plotted.

In the case of Incucyte® Cytotox Dye (Sartorius) staining, cells were exposed to treatments (DMEM + Cytotox Red dye + CPPs) at different concentrations and incubated for 2 h at 37°C with 5% CO_2._ The cells were then imaged using the Incucyte® Live-Cell analysis instrument. Cytotox Red dye dilution: The dye was diluted to a stock concentration of 100 µM in PBS. This was further diluted in full media to yield a final concentration of 250 nM before adding to the cells.

### Measurement of TEER of Caco-2 monolayers

Transepithelial electrical resistance (TEER) represents the barrier integrity and permeability of the Caco-2 monolayers. To measure TEER, cells were seeded and grown on 12-well cell culture inserts (0.4 µM, 1.1 cm, polyethylene terephthalate membrane, cellQART) for a period of 21 days. TEER was measured using Millicell^®^ ERS-2 Voltohmeter (MERS00002), combined with a STX-04 electrode. Final TEER values were expressed relative to the surface area of the inserts (ΩTotal - ΩInsert X cm^2^). TEER values were monitored every day from day 14 until day 21, before, and after permeability experiments. On day 21 of differentiation, TEER values of the monolayers were 600 ± 50 Ω X cm^2^ (mean ± SD; *n* = 48).

### FITC-dextran translocation assay

For Fluorescein Isothiocyanate (FITC)-Dextran permeability experiments, Caco-2 monolayers differentiated for 21 days were transferred to 12-well plates containing 1.5 ml phenol red free DMEM media (pH 7.4) in the basal compartments. For the treatment, medium in the apical compartment was replaced by 0.5 ml phenol red free DMEM medium containing different concentrations of CPPs, and the plates were incubated at 37 °C for 1 h. Post treatment, inserts were transferred to a new 12-well plate containing 1.5 ml fresh phenol red free DMEM and the apical medium was replaced by medium containing 1 mg/ml FITC-dextran 4 (Sigma-Aldrich). The incubation with the permeability marker lasted for 30 min. Samples were collected from the basolateral compartments post incubation and fluorescence was measured using multi-well fluorescent plate reader (Synergy; excitation wavelength: 490 nm, emission wavelength: 520 nm). The concentration of FITC-dextran in the samples was calculated by comparing the relative fluorescence values to the FITC-dextran standard curve. The apparent permeability coefficient (P_app_, cm/s) was calculated according to Eq. 1.

$$P_{app}=\frac{dQ}{dt}\times \frac{1}{\left(A\times C_{0}\right)},$$ (1)

Where dQ/dt is the drug permeation rate (μg/s); a is the surface area of the inserts (cell monolayer) (cm^2^); and C_0_ is the initial concentration at the apical side (μg/ml).

### Bacterial strains and growth conditions

Gut commensal microbial strains used in this study (Table 2) were purchased from American Type Cell Culture (ATCC) and DSMZ, Germany. The growth and screening experiments were performed in modified Gifu Anaerobic Medium (mGAM) (HyServe GmbH and Co.) as all the strains could grow well in this media. mGAM was pre-reduced for a minimum of 1 day in the anaerobic chamber (Coy laboratory products Inc.) before use.

### Broth microdilution assay for determining antimicrobial effects

The antimicrobial effects of CPPs were determined by performing microbroth dilution method. The overnight cultures of the strains were diluted 100-fold in mGAM broth and 100 µL of the diluted cultured were distributed in the 96-well microplate. Next, 100 µL of CPPs in their respective concentrations were added to respective wells. Plates were incubated aerobically (*Escherichia coli* Nissle, *Escherichia coli* K12, and *Saccharomyces boulardii*) or anaerobically (rest of the strains) at 37 °C for 24 h with or without shaking, respectively. Reading of each plate was performed by measuring the optical density (OD) at 600 nm in a microplate reader (Synergy™ H1 BioTek). The final absorbance values were normalized and represented as values between 0 and 1 as a heat map, where 0 being full inhibition and 1 being no inhibition. The MIC was determined as the minimum concentration of the CPPs at which no significant growth of the strain was observed as compared to medium only (blank).

### Statistical analysis

For statistical analysis, Graph Pad Prism software (Graph Pad Software Inc., San Diego, CA, United States) and Microsoft Excel (Microsoft corporation, United States) were used. Data were presented as means ± standard deviation (SD). Analysis of variance (ANOVA) followed by Dunnett’s multiple comparisons test was applied to determine statistically significant difference (*p* < 0.01) as compared to the controls.

## Data Availability

The raw data supporting the conclusions of this article will be made available by the authors, without undue reservation.
