# Supplementary material for: Screening for effective cell-penetrating peptides with minimal impact on epithelial cells and gut commensals in vitro
Source: Front Pharmacol. 2022 Nov 2;13:1049324. doi: 10.3389/fphar.2022.1049324 (PMC9666501; doi:10.3389/fphar.2022.1049324)
Supplement: Supplementary file 1 [file Table1.DOCX]

Supplementary Material

**Table 1:** Minimum inhibitory concentrations (MICs) of CPPs against gut commensal strains

| **Strains** | **Shuffle** | **Penetramax** | **RRL helix** | **PN159** |
| --- | --- | --- | --- | --- |
| Lactobacillus gasseri | NI | NI | NI | 5 |
| Latilactobacillus sakei | 100 | 100 | 50 | 1 |
| Clostridium bolteae | 50 | 100 | 10 | 1 |
| Bifidobacterium longum | 10 | 10 | 5 | 1 |
| Bifidobacterium adolescentis | NI | NI | 100 | 10 |
| Bacteroidetes vulgatus | NI | NI | NI | 10 |
| Bacteroidetes thetaiotaomicron | NI | NI | NI | 10 |
| Escherichia coli Nissle | NI | NI | 50 | 50 |
| Escherichia coli K12 | NI | NI | NI | 5 |
| Saccharomyces boulardii | NI | NI | NI | NI |
